# Isolated Ocular Stevens–Johnson Syndrome Caused by Lymecycline in a Patient with Underlying Ulcerative Colitis

**DOI:** 10.3390/jcm12165259

**Published:** 2023-08-12

**Authors:** Christine M. Bourke, Brendan K. Cummings, Daire J. Hurley, Conor C. Murphy, Sarah Chamney

**Affiliations:** 1 Department of Ophthalmology, Children’s Health Ireland at Crumlin, Cooley Road, Crumlin, D12 N512 Dublin, Ireland; sarah.chamney@cuh.ie; 2 Department of Ophthalmology, Royal Victoria Eye and Ear Hospital, Adelaide Road, D02 XK51 Dublin, Ireland; dairehurley@gmail.com (D.J.H.); conorcmurphy@rcsi.ie (C.C.M.)

**Keywords:** Stevens–Johnson syndrome, cornea, cicatricial conjunctivitis

## Abstract

Stevens–Johnson syndrome (SJS) and the more severe variant, toxic epidermal necrolysis (TEN), are a spectrum of mucocutaneous reactions with potentially devastating ocular consequences. Ocular complications occur in about 70% of patients with Stevens–Johnson syndrome, and 35% continue with chronic disease. We report an unusual presentation of isolated ocular Stevens–Johnson syndrome in a patient with recently diagnosed ulcerative colitis being treated with Infliximab. The case had an insidious and atypical onset and represented a diagnostic dilemma. The diagnosis was more difficult, due to the fact that the inciting agent had long been stopped. Severe bacterial conjunctivitis such as that caused by Chlamydia Trachomatis, Corynebacterium diphtheria, and Neisseria Gonorrhea can cause forniceal shortening and symblepharon; this diagnosis was ruled out with microbiological swabs. A conjunctival biopsy was the key to diagnosis. Treatment involved high-dose IV steroids and dual immunosuppression with Infliximab and mycophenolate mofetil. We sought to employ interventions with the greatest impacts on our patient’s condition. Our experience contributes to the growing evidence supporting intensive ophthalmic management of SJS to prevent long-term vision loss.

## 1. Introduction

Stevens–Johnson syndrome (SJS) and the more severe variant, toxic epidermal necrolysis (TEN), are a spectrum of mucocutaneous reactions with potentially devastating ocular consequences. SJS is defined as having <10% body surface area skin involvement, and TEN is defined as having >30% affected [1]. SJS/TEN is an immune complex-mediated hypersensitivity reaction involving skin and mucous membranes, characterized by epithelial sloughing and blistering from widespread epithelial keratinocyte apoptosis and necrosis [2]. Although SJS can be caused by infections (HSV, mycoplasma) and malignancies, the most frequent precipitant cause is drug exposure. Antiepileptic drugs are strongly associated with SJS, although less strongly associated drugs include sulphonamide antibiotics, allopurinol, nevirapine, and tetracyclines, as we discuss in this case. In some cases, no precipitating factor is identified.

Systemic autoimmune disease is another risk factor for the development of SJS [3,4]. In autoimmune diseases, disturbed regulatory immunity augments cytotoxic reactions, which are associated with the induction of SJS/TEN [5,6].

The condition is fatal in 5% of treated cases and in 15% of untreated cases [7]. Ocular complications occur in about 70% of patients with Stevens–Johnson syndrome [8], and 35% continue with chronic disease [9].

Ocular manifestations range from mild to severe and sight-threatening. Conjunctival ulceration over the tarsal plates is common but may be difficult to confirm, due to lid swelling. Corneal epithelial defects may progress to corneal ulceration with the potential for microbial superinfection. Progressive sight-threatening corneal complications require lifelong management and can result from conjunctival scarring involving the lid margin, tarsus, and loss of the fornix. Late complications may result from damage to the limbal epithelial stem cells, from dry eye disease from the obliteration of the lacrimal ductules and goblet cell loss, and from chronic inflammation or infection, causing corneal opacity and neovascularization.

## 2. Case

We report the case of a 15-year-old male referred by his gastroenterologist for a new onset of “blistering conjunctivitis” in both eyes. There was an associated blurring of vision over the previous 4 weeks. His ocular history was notable for right amblyopia. The background medical history was of ulcerative colitis (UC) and acne.

UC was diagnosed six months prior, based on endoscopic and histological findings. An endoscopy showed a pancolitis mainly involving the rectum showing edematous mucosa, erythema, and mucosal friability. Histology showed that the diffuse superficial chronic active colitis of the mucosa was affected, but the deeper layers of the bowel were spared. The patient was suffering with up to five loose bowel motions per day, with blood in 40%. There was associated urgency and tenesmus. There was no weight loss, no abdominal pain, no oral ulcers, and no perianal findings. Bloods were normal at diagnosis. He was ANA negative.

It was decided to commence 40 mg of oral prednisolone at this stage. Two weeks later, there was an improvement in his symptoms, with one formed stool per day and no blood. Mesalazine 4 g once daily was commenced, and steroids were tapered. The patient was seen three months later once steroids were stopped. He developed a disease flare (three–four bloody stools per day, lethargy, and decreased appetite). At this point, infliximab infusion was started.

Upon presentation to the pediatric eye clinic three months after the commencement of infliximab, the patient complained of painful, red eyes with epiphora and reduced vision. Visual acuity was 6/15 unaided (no improvement with pinhole—NIPH) in the right eye and 6/9 unaided (6/6 with pinhole) in the left eye. Intraocular pressure was 17 mmHg in the right eye and 18 mmHg in the left eye. Refraction of the right eye was +0.25/+1.25 × 81°, and the left eye was −9.00/+6.00 × 87°. A scissoring reflex was seen on the retinoscopy of the left eye. Central corneal thickness was 547 µm in the right eye and 537 µm in the left eye with pachymetry. There was meibomian gland dysfunction, which was worse in the left eye, and a mechanical ptosis from lid swelling. There was significant tarsal conjunctival inflammation on the left lower lid, with an area of ulceration measuring 1 cm with fluorescein uptake. Prominent corneal nerves and moderate superficial punctate epithelial erosions were noted on both corneas, but there was no ulceration. The anterior chambers were quiet, and the lenses were clear. The dilated fundal exam was normal. The systems review was notable for gastrointestinal distress and altered bowel habits. No urinary or respiratory symptoms were present. There was no mucosal inflammation or ulceration in areas other than the conjunctiva and gastrointestinal tract. There was no associated joint pain or rash. Viral (HSV1/2, VZV, Adenovirus) and bacterial swabs (MC + S, Chlamydia, and Gonorrhea) were taken, and the patient was started on Fucithalmic ointment (three times daily), dexamethasone preservative free drops (Dexafree) three times a day, and regular lubricants.

Upon review 3 weeks later, the patient reported worsening symptoms. The visual acuity was reduced to 6/24 (6/18 with pinhole) in the right eye and 6/12 (NIPH) in the left eye. Small central epithelial defects were present in both eyes. The cicatricial conjunctivitis had worsened, and a severe bilateral forniceal shortening of less than 2 mm was noted. All swabs were negative. At this point, the Dexafree was increased to every 2 h, and the patient was referred to a specialist corneal clinic.

## 3. Diagnostic Testing

**Figure 1:**
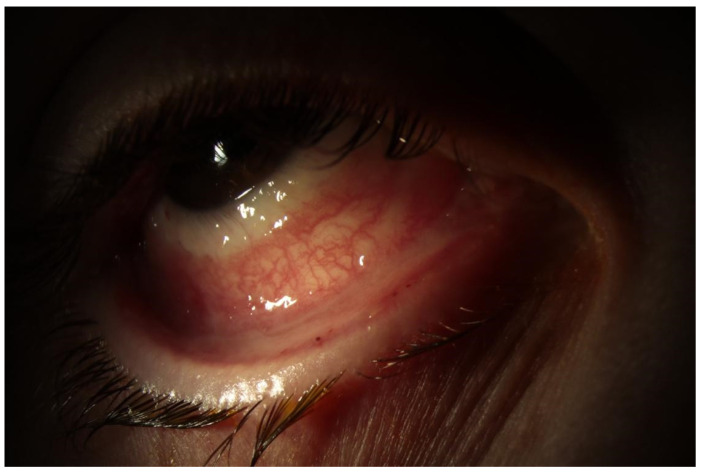
Right eye.

**Figure 2:**
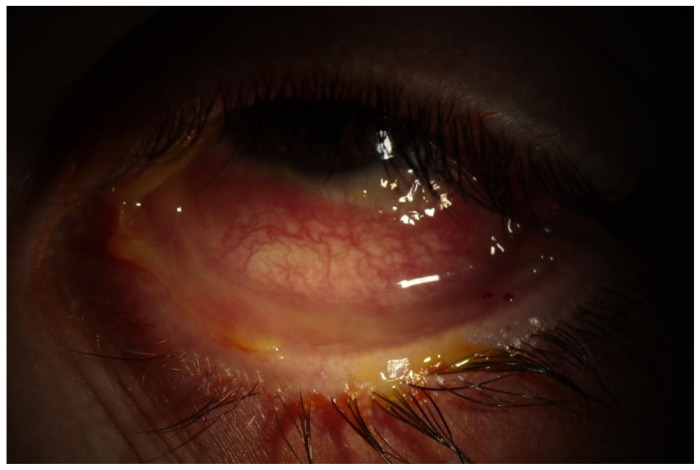
Left eye.

Anterior segment photos were taken in the cornea clinic, which are shown below (Figure 1 and Figure 2). These show diffusely injected conjunctiva bilaterally with forniceal shortening.

A full panel of bloods was taken (FBC, U + E, LFTs, inflammatory markers, Quantiferon, immunoglobulins, serum electrophoresis, and hepatitis A and B titers). Of note, erythrocyte sedimentation rate (ESR) was 23 mm/h [normal: 1–13], and C-reactive protein was 20 mg/L [normal: <10]. All other tests were negative.

The patient underwent a conjunctival biopsy under general anesthesia. Two full thickness biopsies were taken from the temporal bulbar conjunctiva of the left eye (the eye with the more progressive disease). One was sent for histology (in formalin), and the other was sent for direct immunofluorescence (DIF) (in saline).

## 4. Diagnosis and Management

Upon receiving the awaited biopsy results, the differential diagnosis was broad and included mucous membrane pemphigoid, pseudo-Stevens–Johnson syndrome, isolated conjunctival lichen planus, ulcerative colitis-associated mucous membrane pemphigoid, inflammatory bowel disease (IBD)-associated linear IgA disease, and IBD-associated epidermolysis acquisita.

During this time, the patient was admitted for intravenous methylprednisolone 1 mg/kg/day for 3 days, followed by a course of high-dose oral steroids.

The conjunctival biopsy showed an ulcerated mucosa with heavy inflammatory infiltrate composed of lymphocytes, plasma cells, and polymorphonuclear leucocytes. This was present in a band-like manner at the mucosal–submucosal junction. There was no evidence of granuloma formation or vasculitis. The DIF showed no obvious epidermal linings of IgA, IgG, IgM, C3, and fibrinogen, with strong IgM linear deposition around numerous accessory lacrimal tissues. There was no DIF evidence of an ocular cicatricial pemphigoid. The histologic findings were consistent with Stevens–Johnson syndrome.

Following this, a more detailed drug history was carried out, which was notable for a brief course of lymecycline 4 months prior. This was prescribed by the gastroenterology team to treat acne vulgaris. Tetracyclines are a known cause of drug-induced Steven–Johnson syndrome. The patient did not have a rash, fever, or blistering in any other mucous membranes. The inciting medication had already been stopped. A multidisciplinary team became involved in his care, including ophthalmology, general pediatrics, gastroenterology, dermatology, and urology.

Sequential amniotic membrane transplants (AMT) (left eye followed by right eye) were performed, one week apart. A modified symblepharon ring was used to fix the AMT in place, and temporary lateral tarsorrhaphy was made using two 4–0 silk sutures. Autologous serum eyedrops were commenced 1 week later.

A multidisciplinary team meeting took place at this time, involving the ophthalmology and gastroenterology teams. The conclusion of this meeting was to commence the patient on mycophenolate mofetil and to continue infliximab infusions.

## 5. Follow-Up

The patient was seen day one post AMT insertion. At the time, visual acuity was 6/15 in the right eye and 6/9 in the left eye. Complete epithelialization of both ocular surfaces occurred within 1 week of AMT. There was marked trichiasis and distichiasis present in both eyes. The conjunctival edema and hyperemia improved significantly. The corneas showed punctate staining but no focal infiltrates or ulcers. Severe forniceal shortening was present in both eyes. Over the following weeks, the visual acuity improved to 6/12 RE and remained stable at 6/9 LE. The patient is currently taking autologous serum drops (4–6×/day) for both eyes, Hylonight (nocte) for both eyes, mycophenolic acid/Myfortic (720 mg once daily) orally, and infliximab (5 mg/kg every 4 weeks).

The main concern going forward is the conjunctival fibrosis and how this affects his lid position, corneal epithelial defects, and pannus caused by distichiasis and trichiasis. The patient also attends gastroenterology to monitor his IBD, which remains active during this period, but inflammatory markers are now normalizing (most recent CRP 5 mg/L).

## 6. Discussion

There is a broad spectrum of diseases that may lead to cicatricial conjunctivitis, including autoimmune diseases (most commonly mucous membrane pemphigoid), medications, atopy, infections, burns, and malignancies. The work-up requires serological testing and biopsies from the perilesional bulbar conjunctiva for histopathology and direct immunofluorescence. The biopsy was the key to diagnosing this atypical presentation of SJS.

In all cases of SJS, ophthalmic input is essential. Preventive care, including lubrication, topical antibiotics, topical corticosteroids, lysis of adhesions, and, in severe cases, amniotic membrane transplantation, is effective in reducing ocular sequelae.

To date, there are no reports in literature of isolated ocular Stevens–Johnson syndrome. The scarcity of literature involving the pediatric population is likely related to the lower incidence of SJS/TEN in children. In the United States, the estimated incidence of SJS among children is 5.3 per million children per year [10]. In the UK, the incidence rate is 5.76 SJS cases per million, with the highest incidence in patients aged 1–10 years and 80 years or older [11].

Studies of pediatric SJS/TEN are limited [12,13]. Most published cases present with dermatological manifestations alongside ocular signs. Isolated ocular Stevens–Johnson syndrome is rare. These are life- and sight-threatening cutaneous conditions.

Both drug ingestion and underlying autoimmune disease are risk factors for the development of SJS. Generally, the onset is 1–3 weeks after commencing the drug. Our patient had been taking lymecycline for 12 weeks, and the SJS onset was almost 4 months after stopping the medication. This case was complex, as both risk factors had a role to play in disease development. His UC was uncontrolled leading up to disease onset, which was insidious, as is sometimes the case with SJS. This made prompt diagnosis difficult.

The most common ocular manifestation of IBD is anterior uveitis. Other associations include episcleritis and retinal vasculitis. Gastrointestinal manifestations of SJS are known, and in adults, they are associated with significant mortality [14,15,16,17,18]. In addition, the patient was also on infliximab at the time of the onset of SJS. This could have dampened the conjunctival inflammation and led to the subacute progression of the disease over a period of many weeks. We are not aware of any similar reports of this. Biopsy was critical to the diagnosis, as the clinical phenotype was atypical of SJS. It was also possible that anti-TNF therapy modified the clinical phenotype of the disease and led to isolated ocular involvement, which has not been reported to date.

The goals of acute management of the ocular involvement in SJS are rapid control of inflammation, promotion of healing of conjunctival and lid margin ulcerations, prevention of cicatrization of the conjunctiva, and infection prophylaxis [19]. There is no standardized treatment for the prevention of ocular complications, although early AMT is recommended in moderate to severe cases [20,21,22]. Lid taping or lubricant ointment must be used if the patient is anesthetized to prevent exposure keratitis. A topical antibiotic (quinolone) should be used if there is corneal epithelial breakdown. A topical corticosteroid or cyclosporin may help reduce severe conjunctival inflammation. Their use requires caution when there are corneal epithelial defects, as they may mask signs of infection, which can progress rapidly.

Long-term ocular complications are frequent (46%) and include mixed dry eye disease (44%), symblepharon, trichiasis and distichiasis, and chronic ocular surface inflammation (33% each) [23]. One of the most common indications for utilizing an amniotic membrane transplant in pediatric ophthalmology is SJS (67%) [24]. It has been stated that the disease course can be modified if an amniotic membrane graft is performed early in the disease with corneal involvement [20]. Early intervention with AMT may prevent corneal scarring, symblepharon, or ankyloblepharon [21]. It may prevent limbal stem cell failure if performed within 2 days of the onset of symptoms [22].

Episodes of conjunctival inflammation may persist after the systemic disease has resolved or recur at a later stage in a clinical pattern similar to ocular mucous membrane pemphigoid. These recurrences have not been reported to occur in non-ocular tissues; their pathogeneses are obscure [25,26].

In the chronic phase of the disease (>1 month from the onset), treatment focuses on the management of chronic ocular surface disease by eliminating or minimizing toxicity from topical treatments and the introduction of immunosuppressive therapy if there is recurrent inflammation or progressive cicatrization. Severe conjunctival inflammation leads to a sequence of events with devastating consequences without treatment. This begins with the loss of goblet cells and the accessory conjunctival lacrimal glands. Thereafter, an unstable tear film and secondary punctate keratopathy can cause chronic discomfort, photophobia, and reduced vision. Keratinization of the corneal surface results in severe discomfort and loss of vision. Conjunctival inflammation leads to scarring over the upper tarsal plate, which can subsequently cause lid shortening and entropion, resulting in corneal abrasion from trichiasis. Incomplete lid closure (lagophthalmos) is common. Trichiasis, dry eye disease, exposure, and poor surface healing mean that any abrasion can lead to a persistent corneal epithelial defect. This may progress rapidly to corneal stromal melt and perforation, particularly if there is microbial infection. Acute severe inflammation or chronic ocular surface disease may lead to ocular surface failure from the loss of corneal epithelial stem cells.

The other key point is the need for a biopsy in conjunctival inflammatory disease, where the diagnosis is not obvious, and there is an escalation of treatments used, as shown in this case.

This case had an insidious onset. The diagnosis was more difficult, due to the fact that the inciting agent had long been stopped. Severe bacterial conjunctivitis such as that caused by *Chlamydia trachomatis*, *Corynebacterium diphtheria*, and *Neisseria gonorrhea* can cause forniceal shortening and symblepharon; this diagnosis was ruled out with microbiological swabs. The conjunctival biopsy was key to the diagnosis here. The main learning points from this case are that the lack of any characteristic skin rash or mucus membrane bullae (other than the eyes) does not exclude Stevens–Johnson syndrome. Though extremely rare, this may present with findings isolated to the conjunctiva. Another concerning aspect was the patient’s left amblyopia. This meant it was imperative the cicatricial inflammation was managed optimally from the start. Long-term follow-up is required to prevent and address any lid or corneal sequelae that may occur, such as lid margin keratinization, distichiasis, trichiasis, entropion, corneal pannus, and persistent corneal epithelial defects.

## 7. Conclusions

In conclusion, a detailed drug history is essential for all patients with cicatricial conjunctivitis. Although most findings (skin or mucosal) occur within 3 weeks of taking the drug, there may be a longer lag time from drug intake to Stevens–Johnson syndrome development, particularly where the findings are slowly progressive and limited to the eye. We are of the opinion that the underlying UC added an element of diagnostic dilemma to the case and had a role to play in the isolation of phenotype. Maintenance of the ocular surface with preservative-free lubricants, topical steroids, topical antibiotics, and AMT is essential during the active phase of cicatricial conjunctivitis.

We sought to employ interventions with the greatest impacts on our patient’s condition. This was especially important, bearing in mind his right amblyopia. This patient required significant medical and surgical intervention to preserve his vision and control the ocular inflammation. Our experience contributes to the growing evidence supporting the intensive ophthalmic management of SJS to prevent long-term vision loss.

## Data Availability

No new data were created or analyzed in this study. Data sharing is not applicable to this article.
